# Supplementary material for: The diphenylpyrazole compound anle138b blocks Aβ channels and rescues disease phenotypes in a mouse model for amyloid pathology
Source: EMBO Mol Med. 2017 Dec 5;10(1):32–47. doi: 10.15252/emmm.201707825 (PMC5760857; doi:10.15252/emmm.201707825)
Supplement: Supplementary file 1 — Appendix [file EMMM-10-32-s001.pdf]

Appendix: Table of Contents

Appendix Figure S1 and corresponding figure legend page 2

Appendix Figure S2 and corresponding figure legend page 3

Appendix Figure S3 and corresponding figure legend page 4

Appendix Figure S4 and corresponding figure legend page 5/6

Appendix Figure S5 and corresponding figure legend page 7/8

Appendix Figure S6 and corresponding figure legend page 9

Appendix Figure S7 and corresponding figure legend page 10

Appendix Figure S1

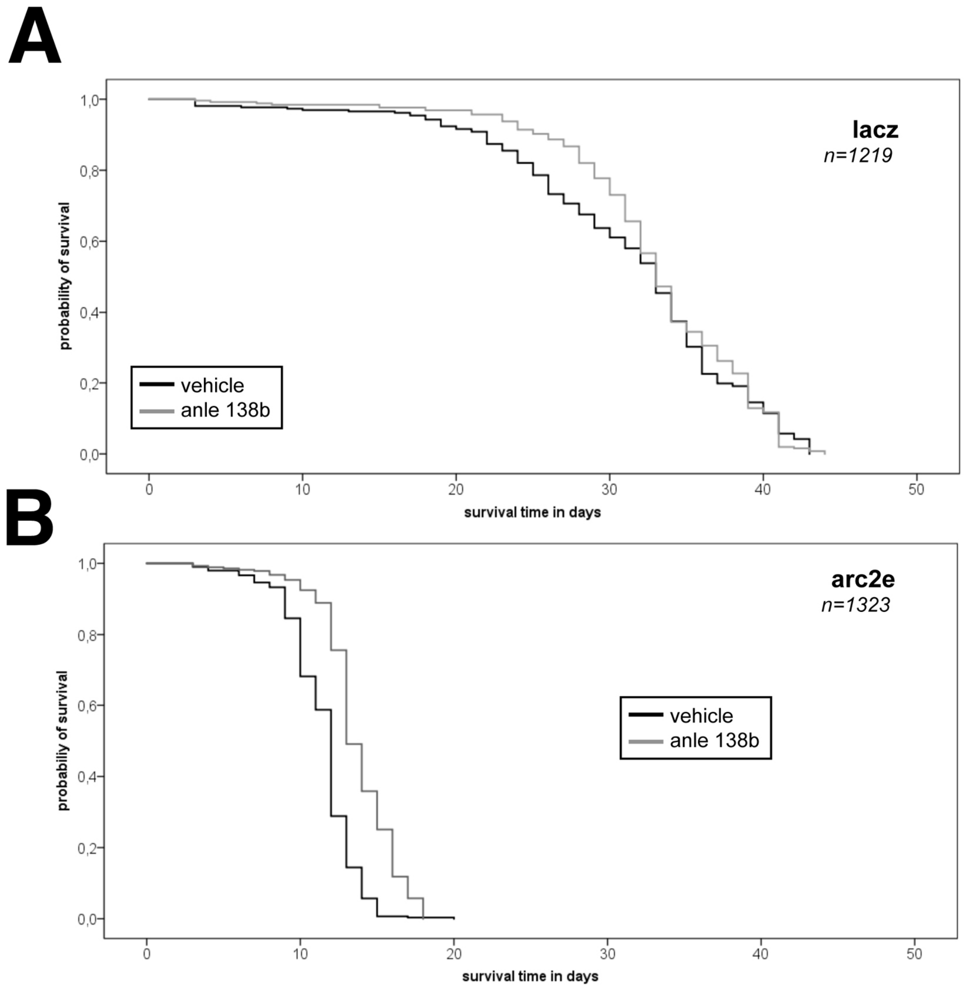

Appendix Fig. S1:.Anle 138b ameliorates Aβ **induced death in a drosophila model.** To test the effect of anle138b on amyloid pathology we used transgenic flies overexpressing Aβ with the arctic mutant under a temperature-dependent promotor (elavGal4/arc2e;tubGal80[ts]). Flies were switched to permissive temperature only after hatching to exclude any developmenteal effect of Aβ expression. A. Flies overexpressing lacz (elavGal4;tubGal80[ts] lacz) were employed as control. Anle138b had no detrimental effect when administered to control flies. B. Consistent with the literature Aβ arc2e expressing flies showed dramatically impaired survival. Treatment with anle 138b treatment showed a significant benefit on survival times compared to the vehicle group (log rank Chi^2=149.41, p = 0.00003 for anle 138b) (lacZ, n = 1219; arc2e, n = 1323).

Appendix Figure S2

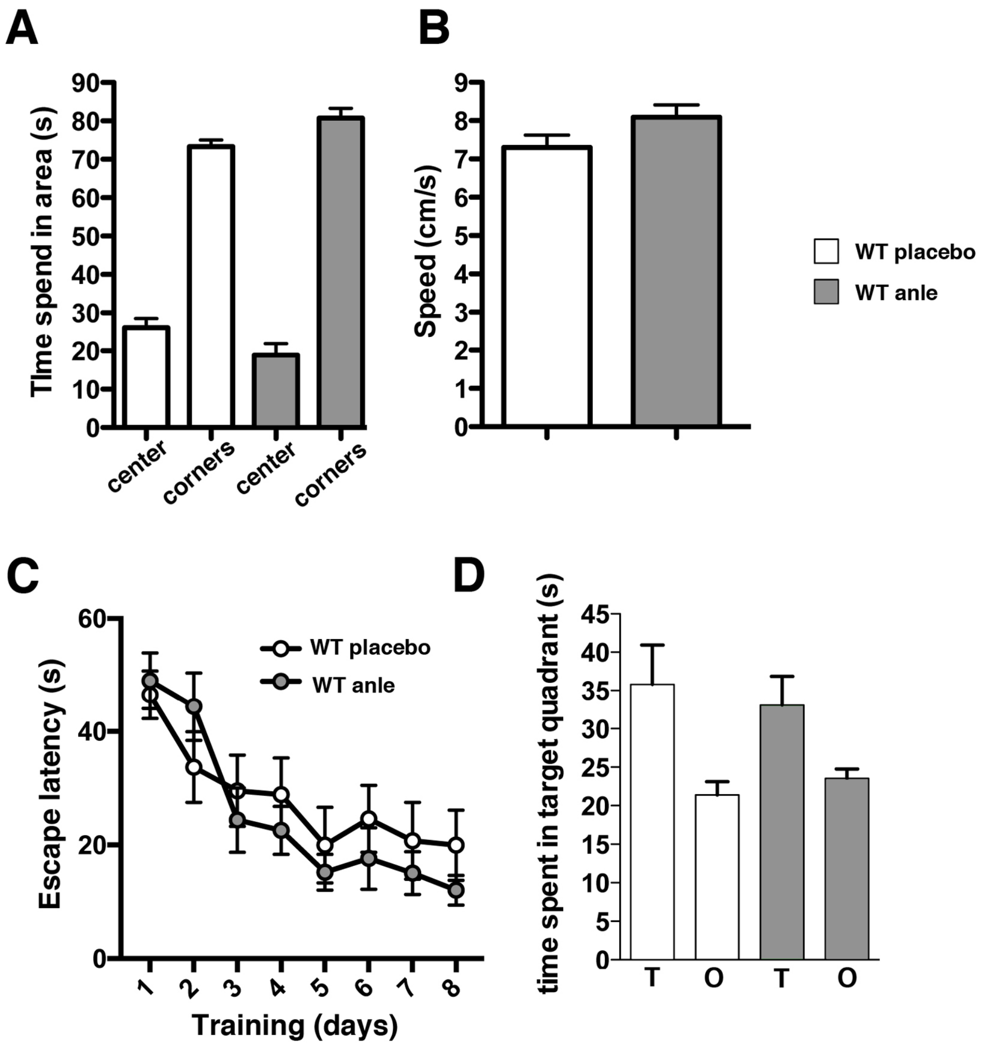

Appendix Fig. S2. Anle 138b does not affect spatial reference memory in wild type mice. Using the experimental paradigm as for the APPPS1ΔE9 mice (pre-plaque group), two-month old wild type mice (n = 8) were fed anle 138b containing food for 4 month. The placebo control group was feed the same food but without anle 138b (n = 8). At 6 month of age mice were subjected to behavior testing. **A.** In the open filed test, the time spent in the center and the corners was similar amongst groups, suggesting that explorative behavior and basal anxiety is not affected in wild type mice treated with anle 138b. **B.** When subjected to the water maze test, swim speed did not significantly differ between the groups. **C.** The escape latency during the water maze training was similar in anle 138b and placebo-treated wild type mice. **D.** The probe test was performed after 8 days of training. The time in the target quadrant (T) was similar in both groups. O; average time spent in other quadrants. Error bars indicate SEM.

Appendix Figure S3

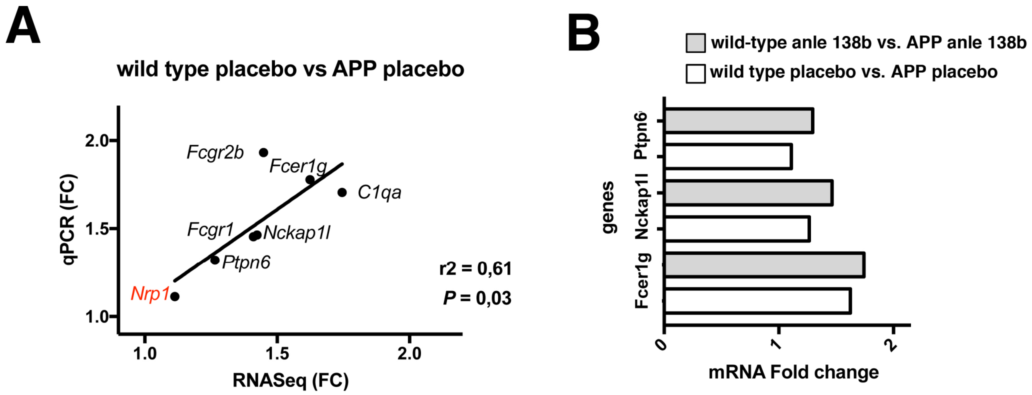

Appendix Fig. S3. qPCR confirms changes in gene-expression changes observed by RNA-sequencing. A. qPCR analysis was performed for randomly selected genes that showed altered expression when comparing RNA-seq data from placebo treated wild type mice vs placebo treated APPPS1ΔE9 mice (APP) in the pre-plaque (Nrp1 gene shown in red) and post-plaque groups (gens shown in black). We observed a significant correlation (Pearson) between RNA-seq and qPCR data (r^2 = 0.06; *P* = 0.03, n = 5/group). Please note that the fold changes in this graph are not presented as absolute values, while the data in supplemental table 1 is shown as log2 values. **B.** For 3 selected genes qPCR analysis was performed in anle 138b or placebo treated wild-type vs. APPPS1ΔE9 mice of the post-plaque group. As observed from the RNA-seq data anle-188b treatment did not affected the expression of these genes. Show are the corresponding fold changes (n=5/group).

Appendix Figure S4

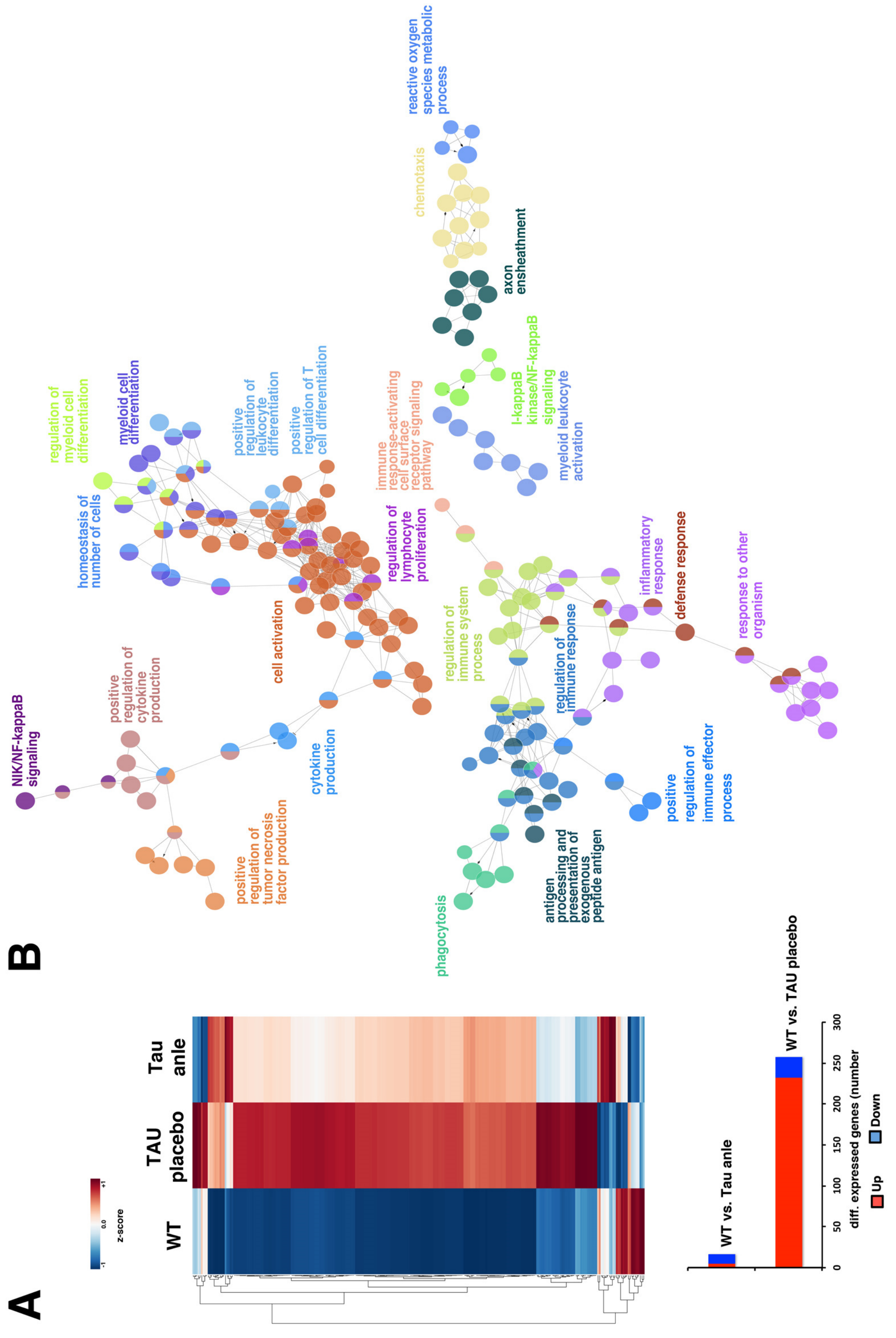

Appendix Fig. S4. Hippocampal transcriptome analysis in anle138b-treated TAUP301S mice. Wagner et al recently reported that treatment of TAUP301S mice with anle 138b from weaning ameliorates disease phenotypes (Wagner et al., Acta Neuropathol. 2015;

PMID:26439832). We obtained hippocampal tissue from the same mice that were killed at the end of the experiment described by Wagner et al., and performed RNA-seq **A.** Upper panel: Heat map showing differentially expressed genes in placebo-treated WT (n=5), placebo-treated TAUP301S mice (n=3) and anle138b-treated TAUP301S mice (n=3). Note that the pathological gene expression profile observed in TAUP301S mice is partially reinstated with treatment of anle138b. Lower panel: Bar graph showing the number of significantly up and down-regulated genes for each comparison. **B.** The genes differentially expressed when comparing placebo-treated wild-type to placebo-treated TAUP301S mice mainly represent pathways linked to neuroinflammation. In line with the fact that only few gene are differentially expressed when comparing placebo-treated WT to anle 138b-treated TAU mice, no significant comparison was identified for this comparisons.

Appendix Figure S5

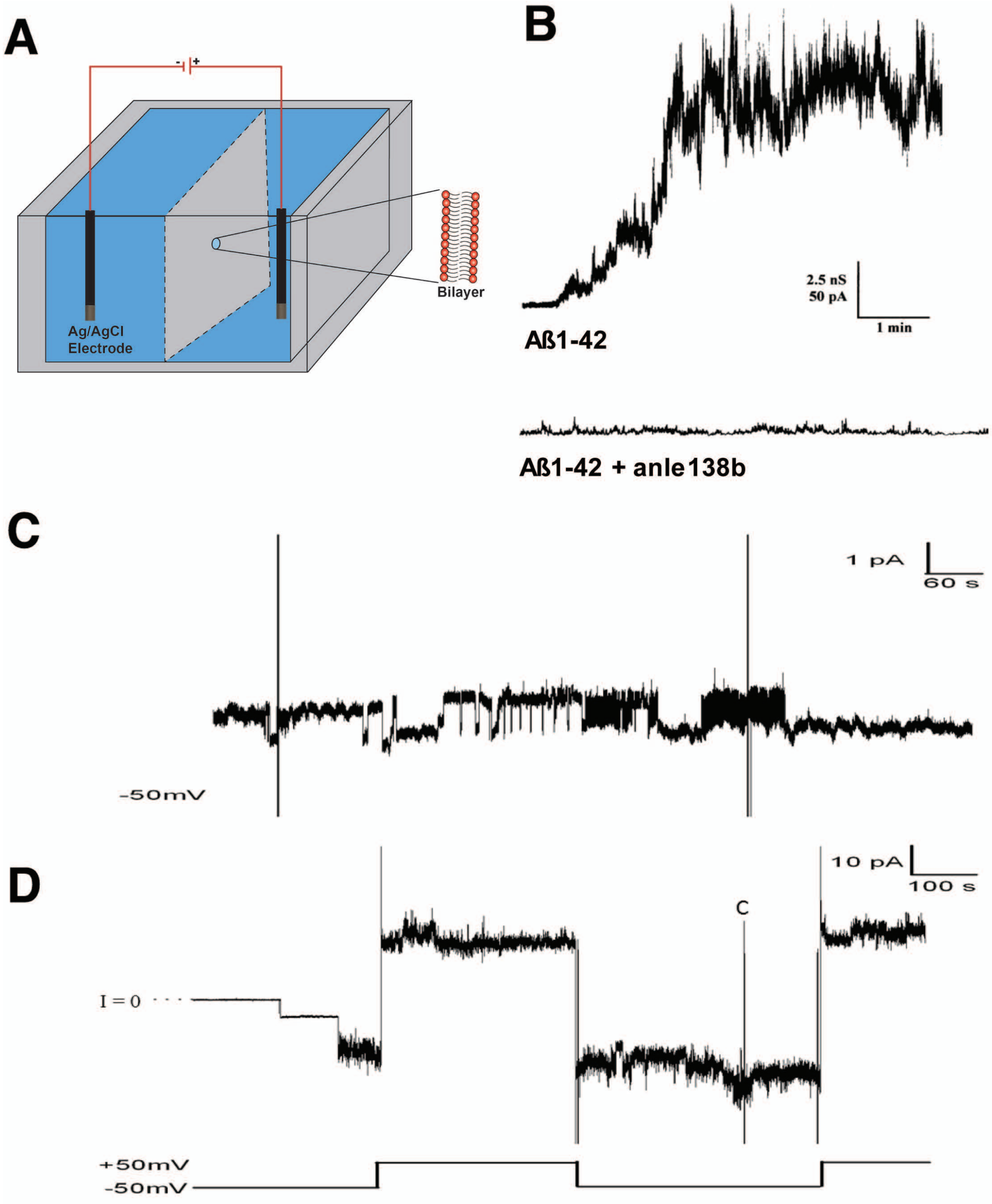

Appendix Fig. S5. Anle138b promotes membrane integrity. A. Schematic representation of the black lipid membrane experiment. **B.** Upper panel: Recording of the conductance increase of an oxidized cholesterol/n-decane membrane after addition of 1 µg/ml Aβ 1-42 to the aqueous phase bathing the membrane. The recording started about 2 minutes after the addition of Aβ 1-42 while stirring. The aqueous phase contained 1 M KCl, pH 6; T = 20°C. The applied voltage was 20 mV. Lower panel: Inhibition of Aβ 1-42-induced membrane conductance with anle138b. The traces show conductance recordings of oxidized cholesterol/n-decane membranes starting about 4 minutes after addition of 1 µg/ml Aβ 1-42 to the aqueous phase bathing the membranes. The membrane forming solution contained 1 mM anle138b, which obviously blocked almost completely the Aβ 1-42-mediated conductance increase. **C.** Control experiments for Fig. 5A, B: Bilayers were formed by the painted technique with a 1:1 (w/w) mixture of DOPS/POPE doped with 10mM Anle138b with respect

to the volume of lipid prior to painting identical to the experiments shown in Fig. 5B. Low conductance events are seen in the presence of anle138b treated membranes. Compared to untreated bilayers, there are fewer simultaneous events and improved membrane stability. **D)** Pore "stacking" was observed in one anle138b treated membrane (shown here) that demonstrated activity modulation, yet at a much reduced level compared to Fig. 5A, namely with a maximum current of around 30 pA . The stacking is likely due to the large area of BLM membranes which could allow individual pores to form in isolated membrane regions where they do not encounter the compound. This could explain the anomaly seen here where a few channels appear to not be interacting with the compound and remain stable in the "open" state. The sensitive nature of BLM recordings allow for the conductance of single channels to be resolved over a large membrane area. We have estimated our membranes to have diameters of 120-180 μm based on membrane capacitances of 110-250 pF and a specific membrane capacitance of 1 $\mu F/cm^2$ for phospholipid bilayers. Over such a vast membrane area a handful of individual channels may not encounter the compound and will therefore exhibit stable behavior in the "open" state.

Appendix Figure S6

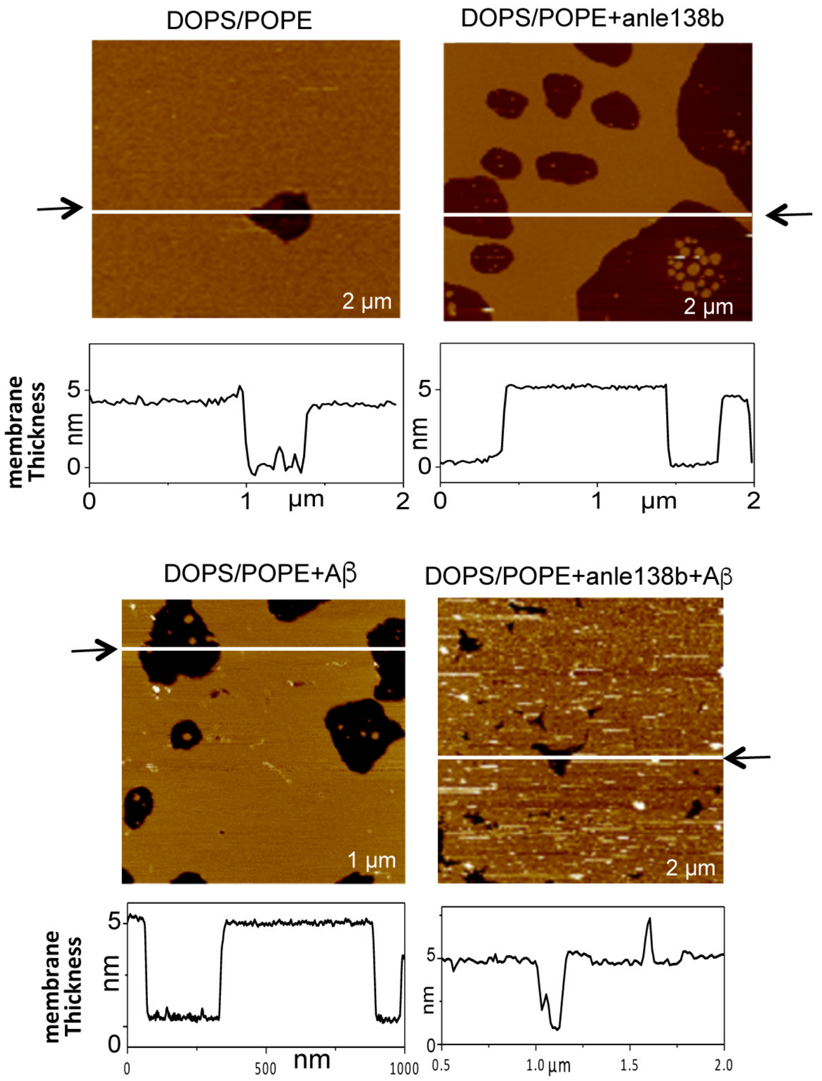

Appendix Fig. S6. AFM and corresponding cross sections showing that the thicknesses of DOPS/POPE (1:1) lipid bilayers (upper left) are not significantly altered by the presence of anle138b (upper right) Aβ (lower left) or anle 138b and Aβ (lower right). The thickness of the membrane is in all cases about 5 nm. The little spots in the images with Aβ are due to Aβ oligomers. The cross sections were obtained across the white lines indicated by arrows in the AFM images.

Appendix Figure S7

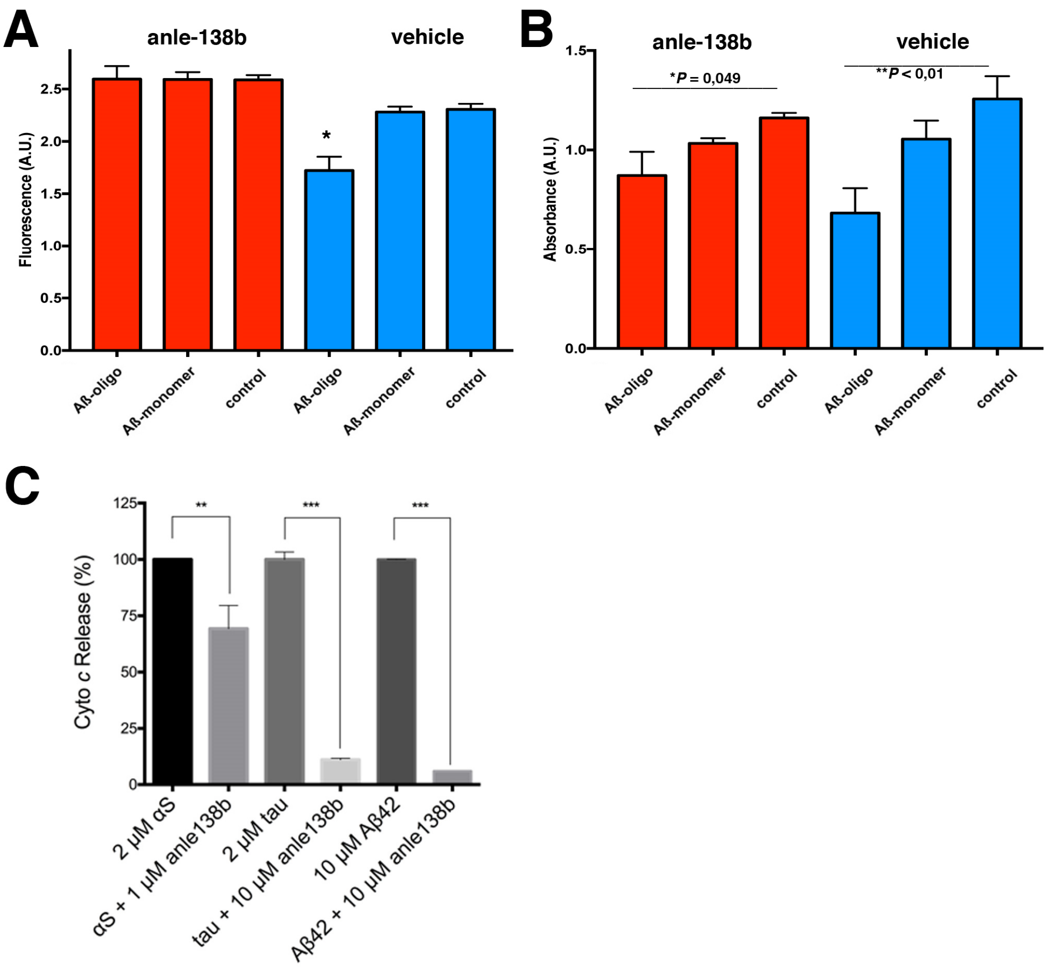

Appendix Fig. S7. Anle 138b reinstates membrane integrity. A. The experiment was performed as described for Fig 5, but in reversed manner. As such hippocampal neurons were first incubated with Aβ monomers or oligomers (10 µM, n=4/group) for 24h before anle138b was added (1 µM). The CyQuant assay was performed 48h later. One-way ANOVA revealed that there was a significant group difference (P = 0.002) and post hoc analysis shows that Aβ oligomers reduce membrane integrity (P = 0.003 vehicle-control vs. vehicle Aβ oligomers while anle138b ameliorated this effect. Error bars indicate SEM. **B.** The experiment was performed as in (A) but cell viability was performed using the MTT assay. One-way ANOVA revealed a significant group difference ($P < 0.05$). Post-hoc analysis revealed that Aβ oligomers significantly impair cell viability in the presence or absence of anle 138b. **C**. Cytochrome release (CCR) assay: Depletion of cytochrome *c* from isolated mitochondria of neuronal SH-SY5Y cells cells was quantified using a colorimetric enzyme-linked immunosorbent assay (Quantikine; R&D Systems, Abingdon, UK). CCR induced by Triton X-100 was taken as reference and set to 100%, as described in Camilleri et al. (2013; PMID:23817009). Efflux of cytochrome *c* from purified mitochondria occurred upon exposure to 2 µM αS oligomers and was comparable to that induced by 2% Triton X-100 (published in Camilleri et al.,2013). Mitochondrial CCR induced by the αS aggregates was however decreased by almost 40% ($p = 0.002$, $n = 2$) when 1 µM anle138b was preincubated with the aggregates. Preincubation with 10 µM anle138b had an even more dramatic effect on tau as well as A□-induced CCR, with an almost complete inhibition ($p = 0.0004$, $n = 2$). Mitochondrial membrane permeabilisation by amyloid aggregates and protection by polyphenols. Error bars indicate SEM.
