# Supplementary material for: The diphenylpyrazole compound anle138b blocks Aβ channels and rescues disease phenotypes in a mouse model for amyloid pathology
Source: EMBO Mol Med. 2017 Dec 5;10(1):32–47. doi: 10.15252/emmm.201707825 (PMC5760857; doi:10.15252/emmm.201707825)
Supplement: Supplementary file 2 — Expanded View Figures PDF [file EMMM-10-32-s002.pdf]

Expanded View Figures

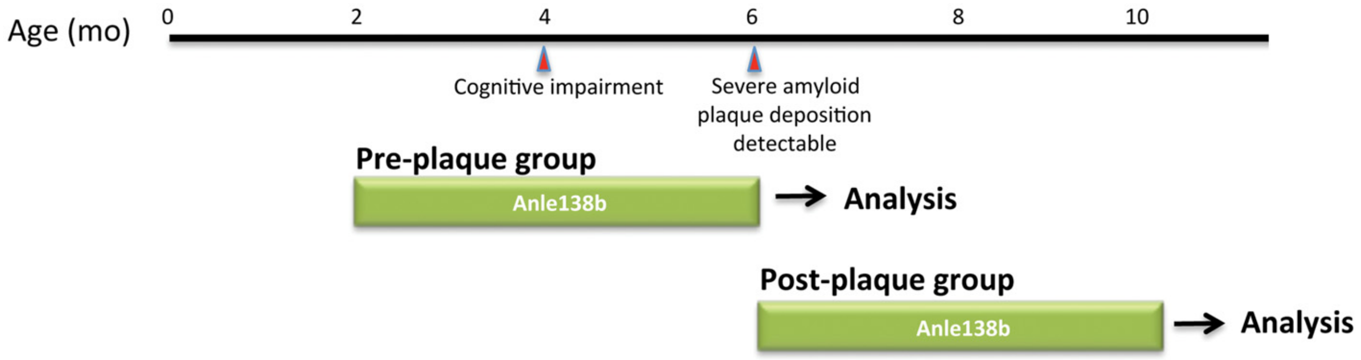

Figure EV1. Experimental design.

Overview of the experimental approach employed for the pre-plaque and the post-plaque group. Red arrows underneath the time line indicate onset of pathology in APPPS1△9 mice.

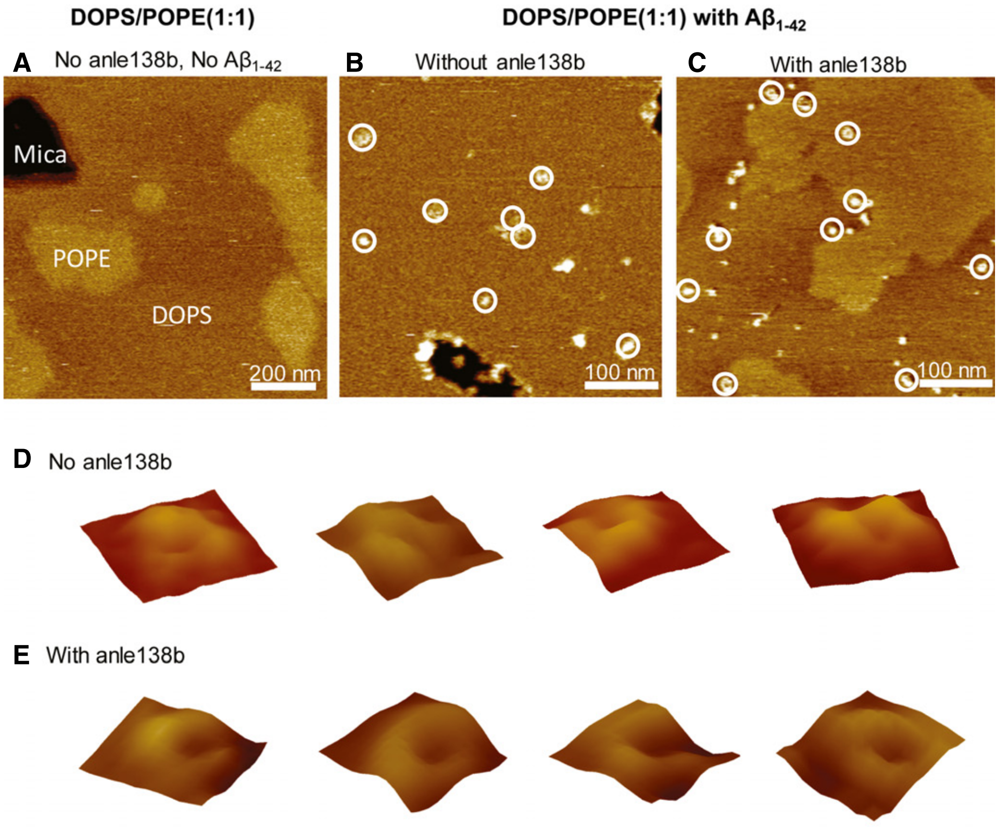

F

	No anle138b	
	Measured diameter	Corrected diameter
Pores (n=18)	13.2 ± 2.7 nm	7.9 ± 2.3 nm
	With anle138b	
	Measured diameter	Corrected diameter
Pores (n=15)	13.8 ± 3.7 nm	8.1 ± 3.3 nm

Figure EV2. Observation of Aβ pores by AFM in anle138b-doped membranes.

AFM tapping mode images showing similar pore structures in membranes with and without anle138b.

A POPE/DOPS (1:1) membranes without compound and not exposed to $A\beta_{1\text{-}42}$ showing lateral phase separation. Scale bar is as indicated and *z* scale is 5 nm.

B Membrane with the same lipid composition as in (A) without compound reconstituted with $A\beta_{1\text{-}42}$ at a 1:60 peptide/lipid molar ratio. White circles represent the position of pore-like structures. Scale bar is 100 nm and *z* scale is 5 nm.

C Membrane with the same lipid composition as above with anle138b (10 mM with respect to lipid volume) and reconstituted with $A\beta_{1\text{-}42}$ (1:60 peptide/lipid molar ratio). White circles represent the position of pore-like structures. Scale bar is 100 nm and *z* scale is 5 nm.

D, E Four representative pores extracted from (B) and (C), respectively. Image size is 23.4 nm × 23.4 nm.

F Table summarizing that Aβ pores show the same dimension in the presence or absence of anle138b. Values are represented as average ±SD.

 © 2017 The Authors
